# The Dose Proportionality of Telcagepant after Administration of Single Oral and Intravenous Doses in Healthy Adult Subjects

**DOI:** 10.1111/j.1753-5174.2010.00031.x

**Published:** 2010-12

**Authors:** Tae H Han, Rebecca L Blanchard, John Palcza, Ashley Martucci, Cynthia M Miller-Stein, Maria Gutierrez, Deborah Panebianco, Ronda K Rippley, Christopher Lines, M Gail Murphy

**Affiliations:** * Merck & Co., Inc.Whitehouse Station, NJ, USA; † Seattle Genetics IncBothell, WA, USA; ‡ Comprehensive Phase OneMiramar FL, USA

**Keywords:** Telcagepant, Migraine, Pharmacokinetics, Dose Proportionality

## Abstract

**Introduction:**

Telcagepant (MK-0974) is a novel, orally active and selective CGRP receptor antagonist being investigated for acute treatment of migraine. Early clinical data suggested greater than dose proportional increases in exposure following oral administration. The aim of the present studies was to definitively characterize the oral and IV dose proportionality of telcagepant.

**Methods:**

Healthy adult subjects were enrolled in two separate open-label randomized dose proportionality studies: 1) single oral dose crossover from 50 to 600 mg (N = 19); 2) single IV dose parallel group from 5 to 250 mg (N = 10 per dose). Blood samples were collected at time points from 0 to 48 hours postdose.

**Results:**

Telcagepant was rapidly absorbed with a T_max_ of approximately 1 to 2 hours after oral administration. The terminal half-life was approximately 8 to 9 hours after IV dosing and approximately 4 to 7 hours after oral dosing. Oral administration of telcagepant resulted in greater than dose proportional increases in exposure, while IV administration resulted in approximately dose proportional increases in exposure.

**Conclusions:**

Telcagepant was generally well tolerated. Oral telcagepant exhibits non-linear pharmacokinetics.

## Introduction

Migraine is a common disease and a leading cause of disability [1]. In the United States, work loss due to migraine is estimated to cost $13 billion [2]. Calcitonin gene-related peptide (CGRP) is a neuropeptide that may play a key role in the pathophysiology of migraine [3,4]. Since CGRP receptor antagonists lack direct vasoconstrictor activity, this new mechanism of action may offer advantages over current triptan treatments, where cardiovascular liabilities are a major perceived risk [5], or may offer another treatment option in patients who do not respond to triptans.

Telcagepant (formerly known as MK-0974) is an orally available, selective and potent antagonist of the human CGRP receptor [6–8]. Telcagepant is currently in development for the acute treatment of migraine, and may have the potential to treat migraineurs with cardiovascular disease. The efficacy and tolerability of telcagepant has been demonstrated in phase 3 clinical trials at doses of 150 and 300 mg [9,10].

Non-clinical assessments of telcagepant in monkeys and rats suggested species dependent non-linear pharmacokinetics following oral doses of telcagepant. Intestinal first-pass metabolism was concluded to play a significant role [11]. A similar observation was made in early clinical studies [12] in which greater than dose proportional increases in exposure following oral administration were apparent. Here, the dose proportionality of telcagepant is formally characterized in a definitive oral dose proportionality study and compared to the results from a second study in which the dose proportionality following IV administration of telcagepant was characterized.

## Material and Methods

### Study Design

Two single-center, open-label, randomized studies were performed. The oral dosing study (Merck Protocol 029) was a 5-period crossover design with doses of 50, 150, 300, 450 and 600 mg. The IV study (Merck Protocol 019) was a 7-panel parallel design with doses of 5, 10, 25, 50, 100, 175, and 250 mg; the study used a parallel design because of dosing limitations imposed by the inclusion of Captisol® in the IV formulation (possible renal toxicity). The oral study was conducted at CPI Comprehensive Neuroscience, Inc., Miramar, FL, USA and the IV study was conducted at CEDRA Clinical Research, Austin, TX, USA. The studies were performed in conformance with legal requirements for the ethical conduct of research in human subjects and were approved by Independent Investigational Review Board, Inc., Plantation, FL (oral study) and IntegReview Ethical Review Board, Austin, TX (IV study). All subjects gave written informed consent.

### Patients

Both studies enrolled healthy, non-smoking adult male and female subjects. Subjects were required to have a body mass index ≤33 kg/m^2^ (oral study) or ≤35 kg/m^2^ (IV study), and females were required to have a negative pregnancy test at screening and agree to remain abstinent or use appropriate double barrier contraception during the study. The oral study enrolled 20 subjects; 14 males ages 25 to 55 years, and 6 females, ages 39 to 43 years. One subject who completed the study but took concomitant medication was excluded from the pharmacokinetic analysis. The IV study enrolled 70 subjects (10 per dose group); 36 males ages 18–44 years and 34 females ages 19–43 years.

### Drug Supplies

Oral telcagepant was supplied in soft gelatin (oral soft elastic) capsules manufactured by Banner Pharmacaps, Inc. The bulk drug used was telcagepant, monopotassium salt, ethanolate, supplied by Merck Research Laboratories. The IV formulation of telcagepant consisted of telcagepant, monopotassium salt, ethanolate infused as a 6 mg/mL solution (containing 35% Captisol®).

### Study Procedures

Both studies were performed in specialist clinical research units. Concomitant medication was prohibited from 48 hours before dosing. For the oral 5-period, crossover study, the order in which the subjects received each dose was randomized according to a computer generated allocation schedule. Each subject received each dose with 240 mL of water at the same time in each period, after an overnight 8-hour fast. There was at least a 7-day washout interval between each treatment period. For the IV study, the treatment group to which subjects were assigned (10 per group) was enrolled sequentially. The study started with the lowest dose group (5 mg) and subsequent groups were dosed incrementally (10, 25, 50, 100, 175, and 250 mg) dependent on the tolerability and pharmacokinetic profile observed in the previous group. For each subject, after an overnight fast, the appropriate volume of IV solution was withdrawn from pre-filled vials and then infused via infusion tubing. Doses were administered at a constant rate of 20 minutes via a syringe-type, rate-controlled, infusion pump. In both studies, plasma samples were collected at predose and at specified time points up to 48 hours following dosing and subject safety was monitored by repeated clinical and laboratory evaluations, including assessment of adverse events.

### Bioanalysis

Plasma samples were analyzed for telcagepant concentrations; details of the bioanalytical methods are published elsewhere [13]. Both analyte and internal standard were isolated from human plasma using solid phase extraction in 96-well format. The extracted analytes were analyzed by high performance liquid chromatography with tandem mass spectrometry. The lower limit of quantitation for telcagepant was 5 nM. The linear calibration curve range was 5 to 5000 nM.

### Pharmacokinetic and Statistical Methods

The (apparent) terminal rate coefficient (λ) was estimated by regression of the terminal log-linear portion of the plasma concentration-time profile. The (apparent) terminal half-life was calculated as the quotient of ln(2) and λ. The area under the plasma concentration versus time curve to the last time point (AUC_last_) was calculated using the linear trapezoidal method for ascending concentrations and the logarithmic trapezoidal method for descending concentrations. The area under the plasma concentration versus time curve (AUC_0-∞_) was estimated as the sum of AUC_last_ and the quotient of the last measured concentration and λ. The maximum concentration observed (C_max_) and the time in which C_max_ was observed (T_max_) were assessed by inspection of the plasma concentration data. For the intravenous study, the actual volume of telcagepant delivered was estimated from the difference in mass of the filled syringe, before and after telcagepant administration. The clearance was calculated as the quotient of the dose and AUC_0-∞_. The volume of distribution at steady-state was calculated as the product of the clearance and mean residence time at steady-state. WinNonlin (Pharsight) 5.0.1 was used for all pharmacokinetic calculations.

For the oral study, the primary assessment of dose proportionality for telcagepant AUC_0-∞_ was performed using the power model, as defined on the log scale below, in which µ is the overall mean, β is the overall slope, S_j_ is the random effect of subject j and ε is the random error:


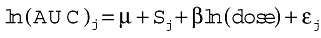


The slope (β) was estimated using a mixed-effects model with dose as a fixed effect and with subject as a random effect. The slope was estimated with an appropriately chosen contrast. On the original scale, the power model is expressed as AUC = α_j_(dose)^β^, with α_j_ = exp(µ + S_j_ + ε_j_). Under this model, exact dose-proportionality is present when the true value of β = 1. The degree of dose proportionality over the dose range was estimated by calculating the 90% confidence interval for the slope.

To better illustrate the effect of the estimated slope, the true increase in AUC_0-∞_ resulting from a 12-fold increase in dose (i.e., over the entire dose range studied) was estimated using the relationship AUC_600-mg_/AUC_50-mg_ = 12^β^, where β was the slope estimated from the power model. The 90% confidence interval for this ratio was also calculated by substituting the lower and upper bound of the 90% confidence interval computed for the slope into the relationship above. Based on the observed data, the method to estimate dose proportionality over the entire range was successively applied to smaller segments (50–450 mg, 50–300 mg) to estimate dose proportionality over other ranges of interest.

Similar analytic methods were used to analyze data from the IV study. Due to the parallel group design, the random effect of subject j was not included in the power model. To better illustrate the effect of the estimated slope, the true increase in AUC_0-∞_ resulting from a 50-fold increase in dose (i.e., over the entire dose range studied) was estimated using the relationship AUC_250-mg_/AUC_5-mg_ = 50^β^, where β was the slope estimated from the power model. The 90% confidence interval for this ratio was also calculated by substituting the lower and upper bound of the 90% confidence interval computed for the slope into the relationship above.

## Results

### Single Oral Dose Pharmacokinetics

**Figure 1:**
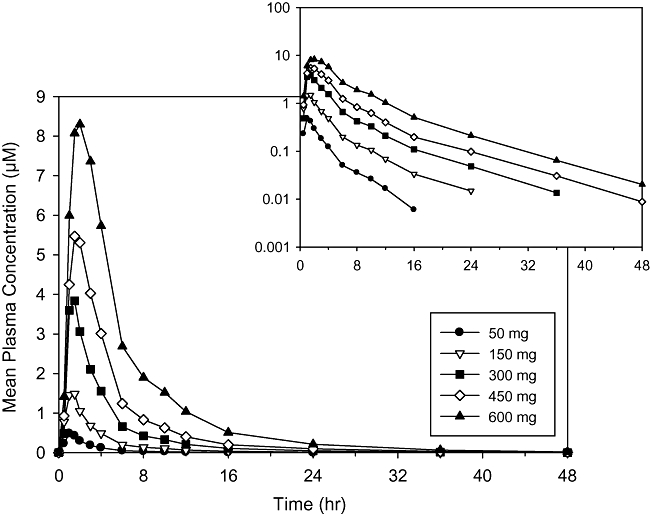
Mean telcagepant plasma concentrations versus time following single oral doses of 50 mg to 600 mg in healthy adult subjects (inset: semilog scale) (N = 19).

Telcagepant was rapidly absorbed with median time to peak plasma concentration (T_max_) ranging from 1 to 2 hours (Table 1). Telcagepant concentrations declined from the peak plasma concentration (C_max_) in a biphasic manner (Figure 1) with an apparent terminal half-life (t_1/2_) of approximately 4 to 7 hours (Table 1). T_max_ was similar across doses. The apparent terminal half-life appeared to increase with dose; however, this may be confounded by the inadequate characterization of the terminal phase at lower doses because the concentration fell below the lower limit of quantification.

**Table 1:** Summary of telcagepant plasma pharmacokinetics following single oral doses in healthy adult subjects (N = 19)

	AUC_0-∞_		AUC_0-4h_		C_max_		T_max_		t_1/2_	
	(µM·h)		(µM·h)		(µM)		(h)		(h)	
Dose (mg)	GM	CV (%)	GM	CV (%)	GM	CV (%)	Median	(Min, Max)	HM	Pseudo SD
50	1.38	44	0.94	45	0.57	45	1.00	(0.50, 1.50)	4.1	1.6
150	5.05	49	3.21	51	1.60	55	1.50	(0.50, 1.50)	6.8	2.6
300	14.03	49	8.31	50	3.89	53	1.50	(1.00, 3.00)	6.5	1.5
450	25.04	41	13.93	38	5.75	37	1.50	(1.00, 3.00)	6.9	2.6
600	48.71	38	23.52	32	9.04	30	2.00	(1.00, 4.00)	6.8	2.2

GM = Model based geometric mean; Min = Observed minimum; Max = Observed maximum; HM = Harmonic mean; SD = Standard deviation; CV = Coefficient of Variation.

**Figure 2:**
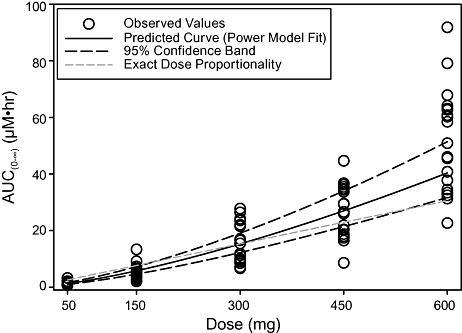
Assessment of dose proportionality for telcagepant AUC_0-∞_ following single oral doses of 50 mg to 600 mg in healthy adult subjects (N = 19).

Table 2 contains point estimates and 90% confidence intervals for the slopes from the power model used to assess dose proportionality of telcagepant AUC_0-∞_, AUC_0-4h_ and C_max_ over the dose ranges of interest. Greater than dose proportional increases were observed in the area under the concentration-time curve estimated to infinity (AUC_0-∞_) and estimated to 4 hours post dose (AUC_0-4h_) over the range of 50 to 600 mg, 50 to 450 mg, and 50 to 300 mg. While the increase in AUC were greater than dose proportional over all ranges evaluated, C_max_ was greater than dose-proportional over the range of telcagepant 50 to 600 mg, but approximately dose-proportional over the ranges of 50 to 300 mg and 50 to 450 mg. The estimated regression line and corresponding 95% confidence band are displayed graphically in Figure 2 for AUC_0-∞_.

**Table 2:** Dose proportionality of telcagepant following single oral doses in healthy adult subjects (N = 19)

Pharmacokinetic Parameter	No. of Doses	Low Dose (mg)	High Dose (mg)	Estimated Slope† (90% CI)	Predicted Fold-Change (90% CI)	Expected Fold-Change with Perfect Dose Proportionality	MSE*
AUC_0-∞_ (µM·h)	5	50	600	1.40 (1.35, 1.46)	32.6 (28.5, 37.2)	12.0	0.0785
	4	50	450	1.32 (1.26, 1.38)	18.2 (15.8, 20.9)	9.0	
	3	50	300	1.28 (1.20, 1.37)	10.0 (8.6, 11.6)	6.0	
AUC_0-4h_ (µM·h)	5	50	600	1.28 (1.22, 1.34)	24.0 (20.7, 27.8)	12.0	0.0953
	4	50	450	1.23 (1.16, 1.30)	14.9 (12.7, 17.4)	9.0	
	3	50	300	1.21 (1.11, 1.30)	8.7 (7.4, 10.2)	6.0	
C_max_ (µM)	5	50	600	1.11 (1.05, 1.17)	15.7 (13.5, 18.2)	12.0	0.0968
	4	50	450	1.07 (1.00, 1.14)	10.5 (9.0, 12.3)	9.0	
	3	50	300	1.06 (0.97, 1.16)	6.7 (5.7, 8.0)	6.0	

* Mean square error on log-scale.

† Exact dose proportionality present when true slope is equal to 1.

### Single IV Dose Pharmacokinetics

**Figure 3:**
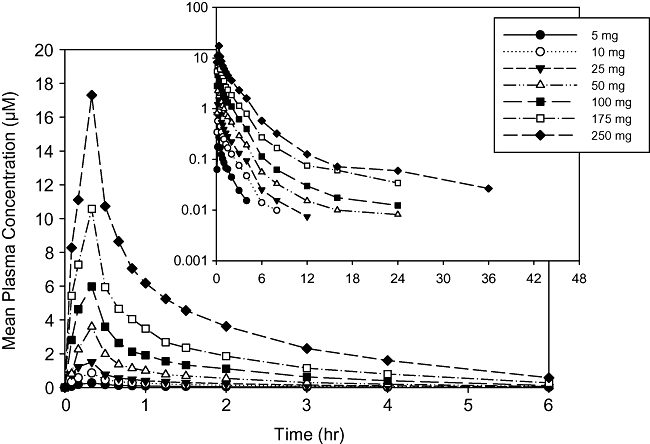
Mean telcagepant plasma concentrations versus time following single IV doses of 5 mg to 250 mg in healthy adult subjects (inset: semilog scale) (N = 10 per dose).

The pharmacokinetics of telcagepant after single IV dose administration of telcagepant to healthy subjects were evaluated over the dose range of 5 mg to 250 mg (Table 3). Following single IV doses, telcagepant exhibited a multiphasic plasma concentration-time profile (Figure 3). The terminal half-life appeared to increase with dose and plateau near the apparent terminal half-life estimate for an oral dose of 300 mg (Table 3). The low half-life estimates from lower doses may not represent the terminal half-life as the concentration of telcagepant was near or below the lower limit of quantitation at those doses. A minor decrease in the clearance was noted with increasing doses, and the volume of distribution increased with dose, though these changes may reflect inaccuracies in the estimation of the terminal phase at the lower doses due to concentrations being below the lower limit of quantitation.

**Table 3:** Summary of telcagepant pharmacokinetics following single IV doses in healthy adult subjects (N = 10 per dose§)

	5 mg IV		10 mg IV		25 mg IV		50 mg IV		100 mg IV		175 mg IV		250 mg IV	
Pharmacokinetic Parameter	GM	% CV	GM	% CV	GM	% CV	GM	% CV	GM	% CV	GM	% CV	GM	% CV
AUC_0-∞_ (µM·h)*	0.28	27	0.85	20	1.54	27	3.90	25	6.98	30	13.03	31	23.73	32
C_max_, C_eoi_ (µM)*	0.26	32	0.81	40	1.47	19	3.43	36	5.67	37	10.32	23	16.38	36
AUC_0-4h_ (µM·h)*	0.25	26	0.75	23	1.34	23	3.16	23	5.83	32	10.35	26	18.64	30
V_d,ss_ (L)†	47.20	26	37.68	38	50.70	27	70.58	31	70.16	55	81.79	39	65.62	41
Cl (L/h)†	32.50	26	23.03	18	27.73	27	21.84	26	24.76	28	22.95	31	18.23	32
t_1/2_ (h) ‡	1.2	0.2	1.6	0.5	2.3	1.2	7.7	9.2	7.8	3.3	8.2	3.2	9.1	1.9

* GM: Geometric mean computed from least squares estimate from an ANOVA performed on the natural-log transformed values. % CV = coefficient of variation.

† Observed geometric means (Non-model based).

‡ Harmonic mean reported for t_1/2_, with pseudo standard deviation.

§ For C_eoi_ at 50 mg IV dose, N = 9. C_eoi_ value for one subject excluded from analysis due to incorrrect infusion time.

**Figure 4:**
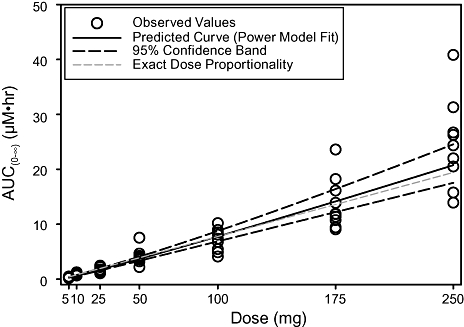
Assessment of dose proportionality of telcagepant AUC_0-∞_ following single IV doses of 5 mg to 250 mg in healthy adult subjects (N = 10 per dose).

Table 4 contains point estimates and 90% confidence intervals for the slopes from the power model used to assess dose proportionality of telcagepant AUC_0-∞_, AUC_0-4h_ and C_max_ over the 5 mg to 250 mg dose range. Following administration of a single IV dose of telcagepant, an approximately dose-proportional increase in AUC_0-∞_, AUC_0-4h_, and C_eoi_ was observed. The estimated regression line and corresponding 95% confidence band are displayed graphically in Figure 4 for AUC_0-∞_.

**Table 4:** Dose proportionality of telcagepant following single IV doses of 5 to 250 mg in healthy adult subjects (N = 10 per dose†)

Pharmacokinetic Parameter	Estimated Slope‡ (90% CI)	Predicted Fold-Change (90% CI)	Expected Fold-Change with Perfect Dose Proportionality	MSE*
AUC_0-∞_ (µM·h)	1.07 (1.03, 1.11)	66.4 (56.5, 78.0)	50.0	0.078
AUC_0-4h_ (µM·h)	1.04 (1.00, 1.07)	57.7 (49.7, 67.0)	50.0	0.067
C_eoi_ (µM)	1.00 (0.95, 1.05)	50.2 (41.8, 60.4)	50.0	0.103

* Mean Square error on log-scale.

† For C_eoi_ at 50 mg IV dose, N = 9. C_eoi_ value for one subject excluded from analysis due to incorrrect infusion time.

‡ Exact dose proportionality present when true slope is equal to 1.

### Safety and Tolerability

Telcagepant was generally well tolerated in both studies. No serious clinical or laboratory adverse experience were reported and no subject discontinued because of an adverse experience. There were no consistent treatment-related changes in laboratory, vital sign, or ECG parameters.

In the oral study, all clinical adverse experiences were mild and resolved. A total of 16 clinical adverse experiences were reported by 4 out of 20 subjects; 1 during a period in which the subject received a single dose of 150 mg, 6 during periods in which subjects received a single dose of 300 mg, 7 during periods in which subjects received a single dose of 450 mg, and 2 during periods in which subjects received a single dose of 600 mg. Those clinical adverse experiences which occurred more than once were constipation, upper respiratory tract infection (one was considered viral), headache, dizziness and nausea.

In the IV study, a total of 29 out of 70 subjects reported 47 adverse experiences. Of the 47 adverse experiences, 17 occurred prior to dosing and 30 occurred on-treatment. Twenty-five of the 47 total reported adverse experiences were related to the IV infusion (e.g., infusion pain). Among the 12 clinical adverse experiences which were on-treatment and were not related to the IV infusion, those which occurred more than once were headache and dizziness.

## Discussion

In the field of migraine therapy, where most medication is self-administered by patients, the availability of an oral formulation is particularly important. Telcagepant is a novel, orally active and selective CGRP receptor antagonist being developed for the acute treatment of migraine. Telcagepant, at doses of 150 and 300 mg of the oral soft elastic liquid filled capsule formulation, demonstrated efficacy superior to placebo in human clinical studies [9,10]. The studies described here were conducted to understand the relationship between dose and plasma exposures following single oral doses of telcagepant capsules ranging from 50 mg to 600 mg and single IV doses of telcagepant capsules from 5 to 250 mg.

Following oral administration, telcagepant is rapidly absorbed with a median T_max_ of 1 to 2 hours and an apparent terminal half-life of 4 to 7 hours [12]. Early clinical studies suggested greater than dose proportional increases in exposure following oral administration, which may indicate changes in clearance or bioavailability [12]. In non-clinical studies, oral dosing of telcagepant in rhesus monkeys was observed to result in much greater than dose proportional increases in exposure. The non-linear pharmacokinetics of telcagepant in rhesus monkeys was attributed to intestinal first-pass metabolism [11]. The initial clinical observations in the context of the non-clinical results prompted the present formal evaluation of the dose proportionality in a definitive oral dose proportionality clinical study and in an IV dose proportionality clinical study.

In the present study, the pharmacokinetics of telcagepant following oral administration to healthy adult subjects were non-linear with greater than dose proportional increases in AUC_0-∞_ and AUC_0-4h_ over all ranges evaluated while C_max_ was greater than dose proportional over the range of 50 to 600 mg. C_max_ was approximately dose proportional over the ranges of 50 to 300 mg and 50 to 450 mg. The possibility that the non-linear oral pharmacokinetics observed in healthy adult subjects were due to interactions at the gastrointestinal level was evaluated by comparing these findings with the dose proportionality of telcagepant following single IV doses.

Following IV administration, approximately dose proportional increases in AUC_0-∞_, AUC_0-4h_, and C_eoi_ were observed with minimal change in the clearance. The terminal half-life appeared to increase with dose, and plateau near the apparent terminal half-life estimate for an oral dose of 300 mg; however, because the concentration of telcagepant was near or below the lower limit of quantitation at lower doses, it is unclear whether this trend is solely due to the pharmacokinetics of telcagepant. Instead, it is likely that the half-life estimates at lower doses do not represent the terminal half-life. A minor decrease in the clearance was observed with increasing doses while the volume of distribution increased with dose. These results suggest that the changes in distribution and elimination are minor, and these changes may not be meaningful in the context of dose proportionality. It is important to understand that due to safety limitations of an excipient in the IV formulation, it was not possible to dose subjects more than once (as in a cross-over study). Therefore, this study used parallel panels of subjects to construct the dose escalation, though it is expected that this design does not impact the conclusions to a meaningful extent.

Telcagepant is metabolized via CYP3A4 and is a substrate of P-glycoprotein in addition to being a time dependent inhibitor of CYP3A4. Both of these proteins are likely to play an important role in the bioavailability of telcagepant and the activity of each protein in the gut wall is theoretically saturable at clinically achievable drug concentrations. Thus, data from the oral dose proportionality study, data from the IV dose proportionality study, and knowledge of the molecular determinants of the disposition of telcagepant, are consistent with the possibility that greater than dose proportional increases in exposure of orally administered telcagepant may be a result of saturable metabolism/efflux of telcagepant in the gut. The relative bioavailabilty of telcagepant increases with dose.

The oral formulation of telcagepant used in the oral dose proportionality study (oral soft elastic liquid filled capsule) was an early formulation. However, this formulation demonstrated bioequivalent exposure, with a dose adjustment, with the anticipated tablet market formulation at two separate doses (140 mg of the tablet compared to 150 mg of the capsule and 280 mg of the tablet compared to 300 mg of the capsule, unpublished data). It is therefore expected that the conclusions about dose proportionality would apply to the anticipated market formulation.

In both studies, telcagepant was generally well tolerated in healthy male and female subjects when given as single oral doses, ranging from 50 to 600 mg, and single IV doses, ranging from 5 to 250 mg. This is in agreement with data from a clinical trial looking at the efficacy and tolerability of single oral doses of telcagepant up to 600 mg in migraine patients [8]. The long term safety and tolerability profile of telcagepant in migraine patients is the subject of ongoing studies.

## Conclusion

In summary, telcagepant is generally well tolerated in young, healthy male and female subjects and exhibits a pharmacokinetic profile suitable for the acute treatment of migraine. The pharmacokinetics of telcagepant after an oral dose were observed to be non-linear with greater than dose proportional increases in exposure. When administered IV, the pharmacokinetics were approximately dose proportional.
